# Digging out the biology properties of tRNA-derived small RNA from black hole

**DOI:** 10.3389/fgene.2023.1232325

**Published:** 2023-10-26

**Authors:** Hengmei Shi, Jiaheng Xie, Shengbin Pei, Danni He, Huyang Hou, Shipeng Xu, Ziyi Fu, Xiaoyan Shi

**Affiliations:** ^1^ Department of Obstetrics and Gynecology, Women’s Hospital of Nanjing Medical University, Nanjing Maternity and Child Health Care Hospital, Nanjing, Jiangsu, China; ^2^ Department of Burn and Plastic Surgery, The First Affiliated Hospital of Nanjing Medical University, Nanjing, Jiangsu, China; ^3^ Department of Breast Surgery, The First Affiliated Hospital of Nanjing Medical University, Nanjing, Jiangsu, China; ^4^ Department of Biomedical Engineering, University of California, Davis, Davis, CA, United States; ^5^ Department of Oncology, The First Affiliated Hospital of Nanjing Medical University, Nanjing, Jiangsu, China

**Keywords:** TRNA-derived small RNA, tsRNA, TRF, tiRNA, non-coding RNAs, non-coding RNA, cancer, biomarker

## Abstract

An unique subclass of functional non-coding RNAs generated by transfer RNA (tRNA) under stress circumstances is known as tRNA-derived small RNA (tsRNA). tsRNAs can be divided into tRNA halves and tRNA-derived fragments (tRFs) based on the different cleavage sites. Like microRNAs, tsRNAs can attach to Argonaute (AGO) proteins to target downstream mRNA in a base pairing manner, which plays a role in rRNA processing, gene silencing, protein expression and viral infection. Notably, tsRNAs can also directly bind to protein and exhibit functions in transcription, protein modification, gene expression, protein stabilization, and signaling pathways. tsRNAs can control the expression of tumor suppressor genes and participate in the initiation of cancer. It can also mediate the progression of diseases by regulating cell viability, migration ability, inflammatory factor content and autophagy ability. Precision medicine targeting tsRNAs and drug therapy of plant-derived tsRNAs are expected to be used in clinical practice. In addition, liquid biopsy technology based on tsRNAs indicates a new direction for the non-invasive diagnosis of diseases.

## 1 Introduction

Many non-coding short RNAs, including microRNAs, lncRNAs, PIWI-interacting RNAs, circRNAs, and others, have been extensively discovered and investigated since the emergence of next-generation sequencing technology (Yang et al., 2022a; Fernandez-Pato et al., 2022; Xie et al., 2022; Xiong et al., 2022). Recently, interest has also grown in the study of tsRNA, an entirely novel category of non-coding RNAs.

As a source of tsRNA, tRNA is one of the non-coding RNAs with the highest abundance. It is typically composed of 70–90 nucleotides and folded into trefoil-like secondary structures consisting of a TΨC loop, an anticodon loop, a D-loop, and a variable loop. With the mutual recognition of the anticodon and the codon, the CCA arm at the 3′-end of tRNA transports a specific amino acid to participate in peptide synthesis with the help of aminoacyl-tRNA synthetase. Therefore, tRNA is an indispensable medium for protein biosynthesis and plays an irreplaceable role in the life activities of individuals. tsRNAs are extensively distributed in bacteria, yeast, mammals, and plants (Mleczko et al., 2018; Gley et al., 2021; Lakshmanan et al., 2021; Wang et al., 2021; Diallo et al., 2022; Sun et al., 2022). Unlike microRNAs, tsRNAs are not transcribed from transcriptome DNA. Instead, they are small molecular RNA fragments generated by tRNA cleavage under the action of a specific ribonuclease in a specific environment. For example, hypoxia, oxidative stress, hunger, heat stress, and inflammatory factors can affect the production of tsRNAs (Green et al., 2020; Geng et al., 2021; Pan et al., 2021; Li et al., 2022b). Even some tsRNAs are sex hormone-dependent (Honda et al., 2015; Zhao et al., 2018). Although not all tRNAs can produce tsRNAs, different tRNAs can generate the same tsRNA (Yang et al., 2022b).

tsRNAs can be categoried into two groups: tRNA halves and tRFs. tRFs can be further subdivided into tRF-3, tRF-5, tRF-1, tRF-2, and i-tRF. Initially, tsRNA has been considered to be a random degradation product of tRNA, but with further research, it has become clear that tsRNA has the potential to play a significant regulatory function in both the physiological and pathological processes of different diseases. Numerous studies have demonstrated that tsRNAs can influence protein translation, gene expression, cell activity, and cell cycle. They may also function as a novel form of epigenetic factor to govern life activities (Shen et al., 2018; Chen et al., 2019; Zhong et al., 2019; Dong et al., 2020). In addition to facilitating the occurrence and progression of neoplastic diseases, including breast cancer, prostate cancer, and liver cancer, tsRNAs also enhance the development of non-neoplastic conditions, such as endometriosis, systemic lupus erythematosus, and renal ischemia-reperfusion injury (Zhu et al., 2019; Farina et al., 2020; Dou et al., 2021; Li et al., 2022a; Li et al., 2022c; Su et al., 2022). Under the guidance of the AGO protein, tsRNAs can target the downstream gene through base pairing and then degrade or inhibit the translation of the target gene, thus playing a microRNA-like function. In HEK293T cells, tRF-3009a can mediate gene degradation and inhibit translation by targeting specific genes via the AGO/GW182 protein complex (Kuscu et al., 2018). tsRNA can also bind proteinto exert biological functions, which distinguishes them from microRNAs (Chen et al., 2019; Boskovic et al., 2020). A multifunctional RNA-binding protein called YBX1 stabilizes various oncogenes and is overexpressed in a number of malignancies. Goodarzi et al. found that tRNATyr, tRNAAsp, tRNAGly and tRNAGlu-derived tRFs competitively bind to YBX1 of the oncogene mRNA 3 UTR (untranslated region) in breast cancer cells. Therefore, tRFs reduce the connection of YBX1 and oncogenic transcripts such as AKT1, EIF4G1 and ADAM8, and then reduce the stability of these oncogenes (Goodarzi et al., 2015). tsRNAs can participate in the initiation stage of cancer by regulating tumor suppressor genes (Zhang et al., 2020) and can also mediate the migration, proliferation and autophagy of tumor cells to participate in the development of diseases (Tong et al., 2020; Zhu et al., 2020). Due to their involvement in a variety of cellular biological processes, tsRNAs can be exploited as a target for precise illness therapy. With the growth of traditional Chinese medicine (TCM) in recent years, the use of tsRNAs derived from Chinese herbal medicine to treat diseases has also emerged (Hu et al., 2022). In addition, the stable existence and wide distribution of tsRNAs in human body fluids has attracted attention for the use of tsRNAs related liquid biopsy techniques, which provide a new direction for non-invasive diagnostic techniques.

This review focuses on the biological function and mechanism of tsRNAs from the perspective of complementary base pairing and protein binding. Additionally, the potential uses of tsRNAs as biomarkers and in the onset, development, and therapy of illnesses are highlighted. Finally, it brings out how tsRNA-based liquid biopsy methods may be used to diagnose illnesses and predict the prognosis of diseases. Using tsRNA as the starting point, we describe in detail the mode of action of tsRNA and the present application of tsRNA in illnesses. This review aims to aid in the in-depth study of tsRNA and to offer theoretical support for the clinical use of tsRNA.

## 2 Origins of tsRNAs

tsRNA is aberrantly expressed in various diseases, especially in cancers. When the cellular microenvironment is altered, cells often have multiple methods to prevent sustained damage from stress factors. Since protein translation requires high energy, cells always take steps to reduce protein production when resources are scarce. These measures often involve tRNA because of its essential role in protein translation (Gupta et al., 2022). tRNA has many modifications that are critical for the structure and function of tRNA (Gupta et al., 2022; Zhang et al., 2023). In healthy cells, tRNA modifications are involved in various biological processes that can help prevent disease. However, tRNA modifications are remarkably different due to the abnormal cellular state of cancer cells. Since cancer cells proliferate at a much greater rate than normal cells, the blood supply is often insufficient, resulting in cells in a state of hypoxia. This stressful environment leads to oxidative stress, which activates multiple tumor-activating signaling cascades. A series of reactions may change the content of modifying enzymes and ultimately affect the modification level of tRNA.

Among the numerous modifications, tRNA methylation plays an irreplaceable role and is essential for tRNA folding and stability (Lin et al., 2018; Endres et al., 2019). TRMT10A is a guanosine 9 tRNA methyltransferase. Under its action, the stability of methylated tRNA^Gln^ and tRNA^iMeth^ is improved. In contrast, TRMT10A deficiency increases the fragmentation of hypomethylated tRNAs, induces oxidative stress, and triggers a pathway of apoptosis in pancreatic β-cells (Cosentino et al., 2018). Moreover, tRNA^Met^ 2′-O-methylation modification mediated by FTSJ1 can also prevent ANG (Angiotensin)-specific cleavage and reduce tsRNA production (He et al., 2020). ALKBH3 is a 1-methyladenosine demethylase. It is highly expressed in various cancers and mediates tumor cell proliferation and disease progression, including lung and colorectal cancers. Demethylated tRNAs are more sensitive to ANG and, in turn, produce more tsRNAs. These functional tsRNAs are involved in ALKBH3-induced tumor progression by preventing apoptosis (Chen et al., 2019). In addition, several RNA modifications can also promote tsRNA biogenesis. An increase in pseudouridine (Ψ) mediated by PUS7 results in increased levels of several types of 5′-tsRNAs (Guzzi et al., 2018). The dysregulation of multiple tRNA-modifying enzymes is associated with various disorders, possibly because many oncogenes and tumor suppressors are involved in regulating tRNA synthesis (Huang et al., 2018). Therefore, abnormal tRNA modification is significant for abnormal tsRNA expression in pathological states.

## 3 Classification of tsRNAs

**FIGURE 1:**
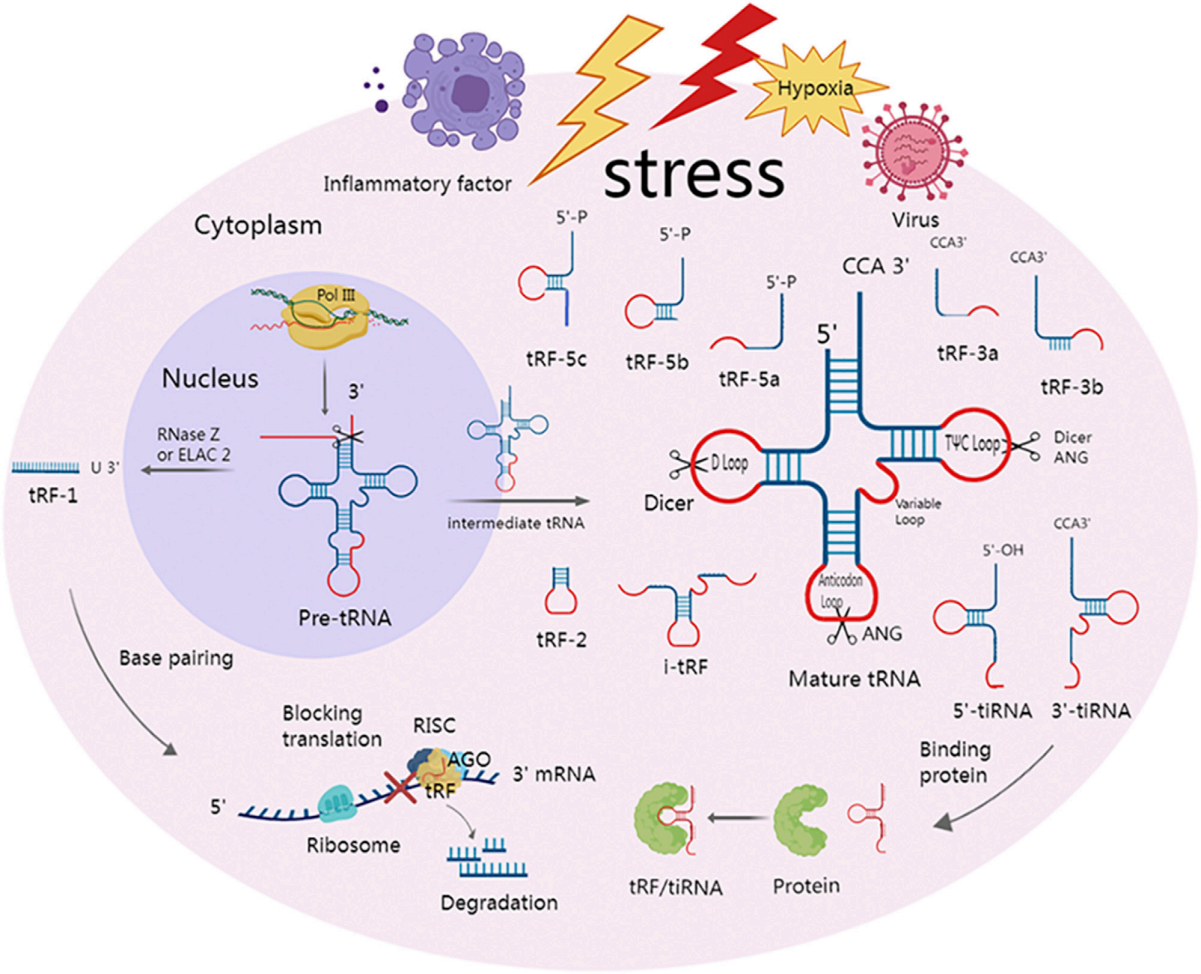
Classification of tsRNAs and two major mechanisms of action. Under various stress conditions, such as hypoxia and viruses, specialized nucleases can cleave precursor and mature tRNA to produce multiple tsRNAs. At the anticodon loop, ANG splits mature tRNA into 5′-tiRNA and 3′-tiRNA. Dicer creates tRF-5 by cleaving the mature tRNA at the D-loop. Dicer or ANG cleaves at the TΨC loop of mature tRNA to form tRF-3. i-tRFs are internal fragments of mature tRNA but do not contain the 3′ and 5′ ends. The anticodon loop and a portion of the stem are present in tRF-2, but the 3′ and 5′ ends are absent. tRF-1 is a small fragment from the 3′ tail of pre-tRNA that is cleaved by RNase Z or ELAC 2. tsRNA can not only bind to downstream target genes by complementary base pairing with the help of RISC, which can cause mRNA degradation or hinder translation but also play a role by directly binding to protein.

Depending on where in a mature or precursor tRNA the cleavage occurs, tsRNA can be categorized into tRNA half and tRF (Figure 1).

### 3.1 tRNA half

tRNA half is derived from mature tRNA and is 30–50 nts in length (Anderson and Ivanov, 2014). These tRNA halves are also known as tRNA-derived stress-induced RNAs (tiRNAs) due to the fact that they are mostly formed under stress by the cleavage of tRNA by ANG However, in yeast, tiRNA is cleaved by a vacuolar RNase T2 family member called RNY1 (Thompson and Parker, 2009; Yamasaki et al., 2009). Recently, it was shown that tiRNAs can also be created in non-stressful circumstances (Kumar et al., 2016). ANG has the ability to split tRNA into 5′-tiRNA and 3′-tiRNA by cutting the anticodon loop. Unlike 3′-tiRNA, which begins at the anticodon loop and ends at the 3′-end of tRNA, 5′-tiRNA begins at the 5′-endof tRNA and terminates at the anticodon loop. It is worth noting that, unlike microRNA, the 5′-terminal of tiRNA is not a phosphate group but a hydroxyl group under the action of ANG or other RNA enzymes (Fu et al., 2009; Yamasaki et al., 2009; Couvillion et al., 2010).

### 3.2 tRF

The length of tRF ranges from 12 to 30 nts, and the cleavage location of precursor or mature RNA allows for classification of tRFs into five different types: tRF-5, tRF-3, i-tRF, tRF-1, and tRF-2 (Anderson and Ivanov, 2014). The D-loop of mature tRNA, or the region between the D-loop and the anticodon loop, is cut by the Dicer to create tRF-5. The three types of tRF-5 are tRF-5a (14–16 nts), tRF-5b (22–24 nts), and tRF-5c (28–30 nt) (Kumar et al., 2014), and the 3′ tail of tRF-5 is usually adenine (Yu et al., 2021). tRF-3 is created by Dicer or ANG cleavage in the TΨC loop of mature tRNA and has a CCA amino acid arm in the tail. Depending on how long it is, tRF-3 can be further divided into tRF-3a (18 nt) and tRF-3b (22 nt). i-tRFs, also called intermediate tRFs, are internal fragments of mature tRNA but do not contain the 3′ and 5′ ends. The anticodon loop and a portion of the stem are present in tRF-2, but the 3′ and 5′ ends are absent. The original tRNA’s 3′UTR, which is cleaved by RNase Z or ELAC 2ELAC2 and has a Poly U sequence, is the source of tRF-1, also known as 3′U-tRF (Lee et al., 2009; Wang et al., 2013; Kumar et al., 2016; Choi et al., 2020).

## 4 Mechanism of action and biological function of tsRNAs

### 4.1 Having microRNA-like biological functions through base pairing

Since tRFs can also be cleaved by Dicer (Kazimierczyk et al., 2022), have a 3′-hydroxyl group and 5′-phosphate group, and their base length is similar to that of microRNA, they can present a microRNA-like mechanism in diseases (Li et al., 2021). In order to create the RNA-induced silencing complex (RISC) and perform biological functions, microRNA can connect to various AGO proteins, such as AGO1, AGO2 and AGO3 (Burroughs et al., 2014). The RISC can also be formed when tRFs bind to the AGO protein. Even some scholars have considered tRFs as a type of microRNA in the past. Induced by RISC, tRFs can target the 3′UTR, or coding region (CDS) of the target gene mRNA through the complementary base pairing principle. This results in mRNA degradation or translational inhibition, which ultimately leads to altered biological functions (Kumar et al., 2014; Huang et al., 2018; Kuscu et al., 2018).

#### 4.1.1 Regulation of rRNA processing

In human and mouse cells, LeuCAG3′tsRNA can interact with the 3′UTRand CDS of ribosomal protein RPS28 mRNA through base pairing, causing mRNA to unfold secondary structure and enhancing RPS28 translation. Inhibition of LeuCAG3′tsRNA leads to apoptosis of rapidly dividing cells in the orthotopic hepatocellular carcinoma model, suggesting that LeuCAG3′tsRNA can exert a specific biological effect (Kim et al., 2019). RPS28 is a crucial part of the 40S ribosome and plays a significant role in the processingsynthesis of the 18S rRNA. Improving RPS28 translation modulates rRNA processing, which has an indirect effect on ribosome biosynthesis (Robledo et al., 2008).

#### 4.1.2 Gene silencing

**FIGURE 2:**
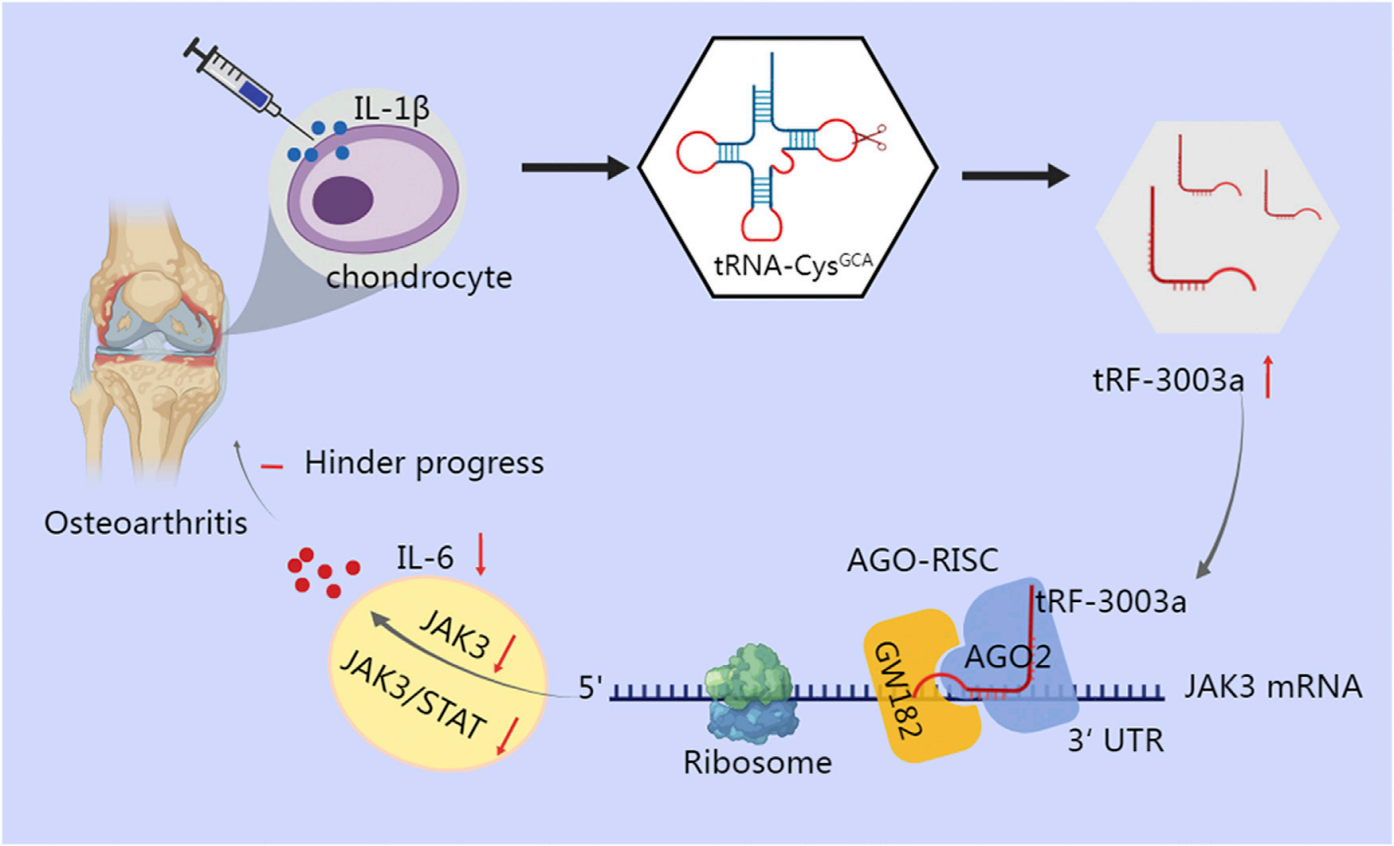
tRF-3003a plays a role in gene silencing through AGO protein in chondrocytes of osteoarthritis. tRNA-Cys-GCA is cleaved and tRF-3003a production is increased when chondrocytes are stimulated with IL-1β. To create RISC, tRF-3003a binds to the AGO2/GW182 protein complex. RISC then targets the 3′UTR of the JAK3 mRNA, silences the JAK3 gene, and subsequently reduces the release of IL-6 by blocking the pro-inflammatory JAK/STAT signaling pathway.

Inflammatory cytokines are crucial for the degradation of cartilage, as well as the onset and advance of osteoarthritis (OA) (Molnar et al., 2021). Green et al. found that stimulation of chondrocytes with IL-1β results in cleavage of tRNA-Cys-GCA and increases production of tRF-3003a. tRF-3003a binds to the AGO2/GW182 protein complex to produce AGO-RISC, which then targets the 3′UTR of the JAK3 mRNA, thereby silencing the JAK3 gene and ultimately down-regulating the expression of IL-6 by inhibiting the JAK/STAT pro-inflammatory signaling pathway (Green et al., 2020) (Figure 2). Similar mechanism has been observed in anterior cruciate ligament cells of OA, where tRF365 silences IKBKB by targeting the 3′UTR of its mRNA, according to a dual luciferase reporter test (Long et al., 2022). Comparatively, in gastric cancer, tRF-24-V29K9UV3IU silences the GPR78 gene under the control of the AGO2 protein in the MKN-45 cell line, ultimately inhibiting cancer progression (Wang et al., 2022).

#### 4.1.3 Decrease protein production

Thrombospondin-1 (THBS1), a member of the thrombospondin-reactive protein family (Huang et al., 2017), is released by activated platelets and has pro-fibrotic and anti-angiogenic effects (Muth et al., 2011; Pal et al., 2016). It is a major participant in the tumor microenvironment and a major physiological activator of TGF-β. tRF-17-79MP9PP, which is only weakly expressed in breast cancer serum and tissues compared to normal tissues and serum, is produced from mature tRNA-Val-CAC and tRNA-Val-AAC. THBS1 is the downstream target of tRF-17-79mp9PP, and this tRF can directly target the 3′UTRof THBS1, reducing its mRNA expression and protein production. This inhibits the THBS1/TGF-β/Smad3 pathway and malignant activity of breast cancer cells (Mo et al., 2021).

Hypoxia is an important tumor microenvironment (Boyd et al., 2021). Under the induction of hypoxia, tRF-20-M0NK5Y93 is downregulated in colon cancer cell line RKO cells. A vital member of the tight junction protein family that is distributed on the surface of cell membranes is claudin-1. It regulates the activation of MMP and is related to tumor metastasis (Chen et al., 2015; Zhao et al., 2015). tRF-20-M0NK5Y93 can directly target the 3′UTR of Claudin-1 mRNA, lowering both its mRNA and protein levels. As a result, the ability of colon cancer cell to express E-cadherin is increased and the invasion of cancer cells is inhibited, and the repressor ZEB-1 is downregulated (Luan et al., 2021). Similarly, when exploring the relationship between tsRNA and Alzheimer’s disease, researchers discovered that tRF-Thr-CGT-003 and tRF-Leu-CAA-004 in APP/PS1 transgenic mice might act on the mRNA of CACNG2 and RYR1 genes and subsequently influence the expression of calcium-regulated associated proteins they encode, such as voltage-gated calcium channel γ2 subunit and RyR1 proteins (Lu et al., 2021).

#### 4.1.4 Regulation of viral infection

In 2009, Yeung and his colleagues made the following finding using high-throughput pyrosequencing: In HIV-1-infected MT4 T cells, a class of 18 nt long non-coding short RNAs generated from tRNAlys3 may enter AGO2-containing RISC and target HIV-1 primer binding site (PBS). It hinders viral replication and functions similarly to microRNA-like RNA interference (RNAi) (Yeung et al., 2009). Likewise, the most active transposons in mice are LTR-retrotransposons, also known as endogenous retroviruses (ERV), which can be suppressed by SETDB1-mediated histone H3 Lysine 9 trimethylation (Matsui et al., 2010; Karimi et al., 2011). However, the PBS, which is required for ERV reverse transcription, can be targeted by 18 nt tRFs to prevent reverse transcription (Schorn et al., 2017).

### 4.2 Functioning by directly binding to protein rather than by base pairing

#### 4.2.1 Influence transcription and reverse transcription

**FIGURE 3:**
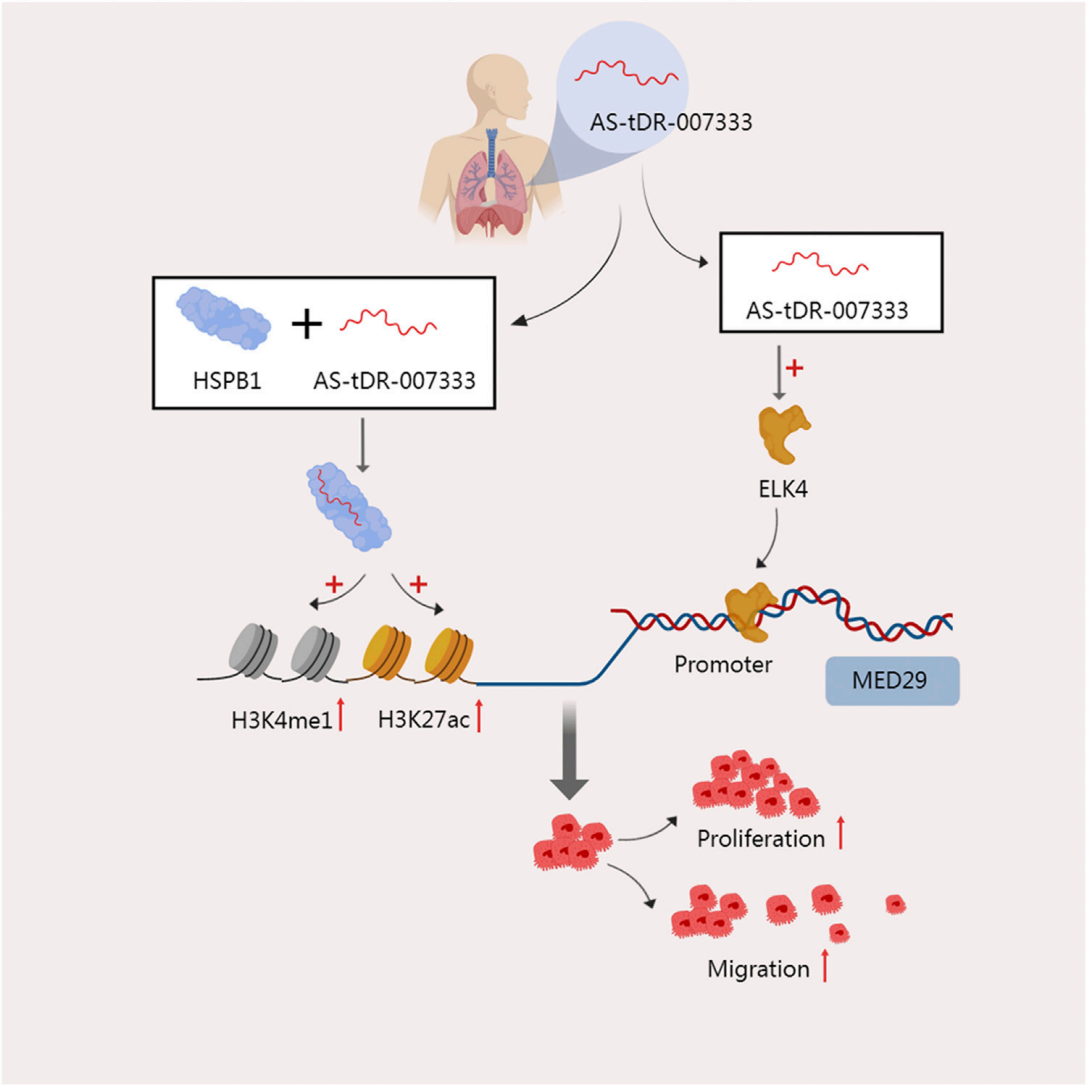
The two modes of action of AS-tDR-007333 jointly improve the malignant potential of NSCLC cells. On the one hand, AS-tDR-007333 binds specifically to the HSPB1 protein and causes HSPB1 to elevate H3K4me and H3K27ac in the MED29 promoter, promoting the transcription of MED29. On the other hand, As-tDR-007333 fosters the production of the transcription factor ELK4, which causes ELK4 to engage with the MED29 promoter and enhances MED29 transcription.

In tissues, plasma, and cells of non-small cell lung cancer (NSCLC), Yang et al. discovered that AS-tDR-007333, an unannotated tRF in the tsRNA database, is highly elevated. High expression of this RNA is also linked to a worse prognosis for patients (Yang et al., 2022b). One of the heat shock protein family members, HSPB1, is overexpressed in response to stress and is found in a number of malignancies (Wu et al., 2017; Yun et al., 2019). It promotes cell proliferation by accelerating the cell cycle and improve the ability of cell migration and invasion (Shiota et al., 2013). The direct interaction between AS-tDR-007333 and HSPB1 was discovered by mass spectrometry analysis and an RNA pulldown test. HSPB1 stimulates MED29 transcription by increasing H3K4me and H3K27ac in the MED29 promoter. Additionally, AS-tDR-007333 may also increase the expression of the transcription factor ELK4, which encourages ELK4 to interact with the MED29 promoter, and subsequently improve the transcription of MED29. These two mechanisms work together to increase the malignant potential of cancer cells’ high rates of proliferating and migrating (Yang et al., 2022b) (Figure 3). The binding of tsRNA to protein can also function at the level of reverse transcription. In mouse embryonic stem cells (mESCs), the 28-nt tRF-Gly-GCC derived from tRNA-Gly-GCC directly binds to the 50 kD hnRNPF/H protein, which is a strong repressor of the endogenous reverse transcription factor MERVL, thus inhibiting the gene expression of MERVL (Boskovic et al., 2020).

#### 4.2.2 Influence protein modification

The most typical type of pancreatic cancer is called pancreatic ductal adenocarcinoma (PDAC). PDAC has a very poor prognosis because there are no early detection tools and the disease is resistant to conventional, targeted, and immunotherapeutic treatments (Taherian et al., 2022). To create an alternative splicing complex in PDAC cells, phosphorylated heterogeneous nuclear ribonucleoprotein L (hnRNP L) preferentially attaches to the protein DDX17. This activated hnRNP L-DDX17 complex cuts the precursor mRNA of mH2A1 into mH2A1.2, which promotes cell invasion, and also shares the precursor mRNA of Caspase 9 to generata generating Caspase 9b, which inhibits cell apoptosis. tRF-21-VBY9PYKHD derived from tRNAGlyGCC could directly bind to Ser52 of hnRNP L, preventing the phosphorylation of hnRNP L by AKT2/AKT1, thereby inhibiting the formation of hnRNP L-DDX17 complex, ultimately inhibiting the invasion ability of cancer cells and promoting their apoptosis. However, in the chronic inflammatory microenvironment of PDAC (Padoan et al., 2019), upregulated leukemia inhibitory factor (LIF) and interleukin-6 (IL-6) can inhibit the expression of tRF-21-VBY9PYKHD through the splicing factor SRSF5. As a result, the inhibitory effect of tRF-21-VBY9PYKHD on tumor cells is diminished, which eventually promotes the progression of cancer (Pan et al., 2021).

#### 4.2.3 Sequester proteins and inhibit translation

tsRNAs can bind specifically to RNA-binding proteins and isolate related proteins in a manner that does not depend on AGO proteins, ultimately affecting translation. Cho et al. examined the small RNA profile of the hepatocellular cancer cell line Huh7 and discovered a group of tRFs produced from pre-tRNA 3′ trailers. Of these tRFs, tRF_U3_1 derived from tRNA2-Ser (TGA) negatively regulates translation which is carried out by the internal ribosome entry site (IRES) of the HCV virus. Through its 3′ U-tail, tRF_U3_1 selectively binds to the nuclear-cytoplasmic shuttling protein La/SSB, isolates it in the cytoplasm, and prevents it from attaching to the stem-loop IV of IRES (Pudi et al., 2003), ultimately inhibiting IRES-mediated translation to resist RNA virus infection (Cho et al., 2019). During the course of a person’s development, cell differentiation is a very dynamic process, and numerous studies have demonstrated the importance of microRNAs in controlling gene expression to promote differentiation (Jiao et al., 2021; Naqvi et al., 2022). However, whether tsRNA is involved in cell differentiation is unclear. Krishna et al. used retinoic acid (RA) to induce mESC to differentiate and discovered that a special type of functional 5′-tsRNAs was enriched in a differentiation-dependent manner. In particular, tsGlnCTG can sequester Igf2bp1 protein from the c-Myc mRNA-protein complex and competitively combine with it. The translation of the pluripotency-promoting factor, c-Myc, is impacted by the tsRNA’s binding to protein, which also impacts transcriptional stability. This research explains the method by which tsRNAs control the differentiation of mESCs (Krishna et al., 2019).

#### 4.2.4 Enhance protein stability

When compared to adjacent tissues, next-generation sequencing revealed that a specific type of 5′-tRNA half, tiRNA-Gly, is abundantly expressed in papillary thyroid carcinoma (PTC) tissues. tiRNA-Gly binds specifically to the UHM domain of the RBM17, which is a splicing-associated RNA-binding protein in the PTC cell line K1 cells. This interaction prevents the ubiquitin/proteasome-dependent degradation of RBM17, allowing it to move from the cytoplasm to the nucleus and increasing the quantity of its protein production. This form of action encourages RBM17-mediated splicing of the exon 16 of MAP4K4 mRNA, which results in the phosphorylation of downstream signaling pathways and eventually increases the capacity of cells to migrate malignantly (Han et al., 2021).

#### 4.2.5 Influence signaling pathways

The interaction between tsRNA and protein in the signaling pathway can exert an inhibitory effect on the signaling pathway. As a common digestive tract tumor, gastric cancer has a global incidence of about 1,089,103 cases, and about 769,000 people die from gastric cancer every year, which ranks as the third-most frequent cause of cancer death (Sung et al., 2021). Since more than 50% of patients are diagnosed after cancer has already spread (Hogner and Moehler, 2022), it is urgent to find the pathogenic mechanism of gastric cancer. According to Cui et al., tRF-60:76-Val-CAC-2 is upregulated in the tissues and cells of gastric cancer (GC), and the degree of its expression is positively correlated with the tumor size and the depth of invasion. The results of the RNA pulldown assays they conducted later revealed that biotinylated tRF-60:76-Val-CAC-2 directly pulls down the chaperone molecule EEF1A1 in AGS cells, mediates its translocation to the nucleus, and promotes its interaction with nuclear MDM2 protein (Cui et al., 2022). The tumor suppressor protein p53 can be targeted by MDM2, an E3 ubiquitin ligase, which can mediate its ubiquitination to contribute to the development of cancer (Amato et al., 2009; Sehdev et al., 2014). This provides new molecular targets for the treatment of cancer. In the process of exploring the relationship between tsRNAs and neurodegenerative diseases, some scholars have found a similar mode of action in the p53 signaling pathway. CLP1 is the first mammalian RNA kinase to be discovered (Hanada et al., 2013). The mutations in human CLP1 are linked to type 10 pontocerebellar hypoplasia. Among tRNA fragments accumulated in CLP1 mutants in the SH-SY5Y human neuroblastoma cell line, 5′Tyr-tRF is potentially toxic to neuronal differentiation. In the zebrafish model, when 5′Tyr-tRF, arising from CLP1 gene mutation, was injected into zebrafish embryos, neuronal defects could also be observed (Inoue et al., 2020). Through the pull-down assay, the researchers demonstrated that biotinylated 5′Tyr-tRF can bind pyruvate kinase M2 (PKM2) tightly and cause neuronal cell death in a p53-dependent manner (Table 1).

**TABLE 1:** Biological functions corresponding to two modes of action of tsRNAs.

Mechanism of action	Function	Disease	Example of tsRNA(s)	Effect	Ref. Number
Complementary base pairing	Regulation of rRNA processingribosome biosynthesis	-	LeuCAG3′tsRNA	-	Kim et al. (2019)
	Gene silencing	Osteoarthritis	tRF-3003a, tRF365	suppress, suppress	Green et al. (2020), Long et al. (2022)
		Gastric Cancer	tRF-24-V29K9UV3IU	suppress	Wang et al. (2022)
	Decrease protein production	Breast Cancer	tRF-17-79MP9PP	suppress	Mo et al. (2021)
		Colorectal cancer	tRF-20-M0NK5Y93	suppress	(Luan et al.)
		Alzheimer’s disease	tRF-Thr-CGT-003	promote, suppress	Lu et al. (2021)
			tRF-Leu-CAA-004		
	Regulation of viral infectionRNA reverse transcription	Viral infection	18 nt tRFs	suppress	Yeung et al. (2009), Schorn et al. (2017)
Binding protein	Influence transcription and reverse transcription	Non-small cell lung cancer	AS-tDR-007333	promote	Yang et al. (2022b)
		-	tRF-Gly-GCC	-	Boskovic et al. (2020)
	Influence protein modificationPost-transcriptional modification	Pancreatic ductal adenocarcinoma	tRF-21-VBY9PYKHD	suppress	Pan et al. (2021)
	Sequester proteins and inhibit translation	Hepatocellular carcinoma	tRF_U3_1	suppress	Cho et al. (2019)
		-	tsGlnCTG	-	Krishna et al. (2019)
	Enhance protein stability	Papillary thyroid carcinoma	tiRNA-Gly	promote	Han et al. (2021)
	Influence signaling pathways	Gastric Cancer	tRF-60:76-Val-CAC-2	promote	Cui et al. (2022)
		Neuroblastoma	5′ Tyr-tRF	promote	Inoue et al. (2020)

## 5 Research and application of tsRNA in diseases

In recent years, an increasing number of studies have demonstrated that tsRNAs are functional fragments that play crucial biological roles in the psychological and physiological processes of numerous disorders rather than being the random breakdown products of tRNAs. tsRNAs can influence the occurrence, development, and treatment of various diseases as well as predict disease occurrence and prognosis.

### 5.1 Occurrence of disease

In the early stage of cancer, protooncogenes and antioncogenes can positively and negatively regulate tumor formation. tsRNAs can either promote or inhibit cancer development by lowering tumor suppressor gene expression or decreasing tumor suppressor gene silence. There are different tsRNAs with opposite biological functions in the same disease. tRF-3019a and tRF-3017A are both highly expressed in the gastric cancer cells. The former silences the tumor suppressor gene FBXO47 by targeting the 3′UTR of its mRNA through the AGO2 protein, while the latter silences the tumor suppressor gene NELL2 through the RISC complex (Tong et al., 2020; Zhang et al., 2020). These two kinds of tRFs collaborate to increase the possibility of gastric cancer by acting on different tumor suppressor genes through a method similar to microRNAs. Nucleolin (NCL), located primarily in the nucleus, is an essential protein for cellular life but also a gateway for viruses, bacteria and toxins to enter the human body (Tonello et al., 2022). The RNA-binding protein NCL, which is abundantly expressed in breast cancer, can bind to the p53 mRNA and prevent p53 from being translated (Takagi et al., 2005; Chen et al., 2012). NCL can also compete with p53 mRNA to bind 3′-tRNAGlu-derived tRF3E to form the NCl-tRF3E complex, thus weakening the silencing effect of tRF3E and NCL on p53. Unfortunately, tRF3E is only expressed in the normal breasts but not in breast cancer (Falconi et al., 2019). On the contrary, ts-112 has oncogenic potential due to its targeting of the tumor suppressor RUNX1 to encourage the growth of breast cancer cells (Farina et al., 2020). By binding NCL and RUNX1, respectively, tRF3E and ts-112 perform an antagonistic role in the beginning stage of cancer formation.

### 5.2 Progression of disease

tsRNAs can influence the progression of diseases from four aspects: proliferation and apoptosis, migration and invasion, inflammation and angiogenesis, and autophagy.

A typical side effect of radiotherapy for thoracic malignant tumors is radiation-induced lung injury (RILI), which often results in a poor prognosis. With X-ray stimulation, bronchial epithelial cells can produce more tRF-Gly-GCC, which in turn can produce more reactive oxygen species (ROS) and induce oxidative stress, leading to the development of RILI. Transfection of tRF-Gly-GCC mimic may reduce cell proliferation, enhance cell death, and eventually promote the formation of RILI through PI3K/AKT and FOXO1 signaling pathways in healthy human lung bronchial epithelial cells (BEAS-2B) (Deng et al., 2022). The epithelial-mesenchymal transition (EMT), which mediates tumor metastasis and dissemination, is a crucial stage in invasion and metastasis. When Gly-tRF binds to the 3′UTR of NDFIP2 mRNA, NDFIP2 mRNA levels drop while the amount of phosphorylated AKT rises, activating the AKT signaling pathway in HCC. Activated AKT signaling enhances tumor cell migration and EMT, speeding up tumor growth by allowing cancer cells to invade nearby tissues (Zhou et al., 2021). As a stressor, inflammatory factors can stimulate tRNA cleavage to produce different types of tsRNAs. However, different tsRNAs can also regulate the content of inflammatory factors. Li et al. discovered that the expressions of the inflammatory factors IL-6, IL-1β, IL-10 and TNF-α are greatly reduced, and the formation of new blood vessels is significantly reduced, when tRF-Leu-AAG-001 is knocked down in the exosomes of mast cells in endometriosis. According to this finding, tRF-Leu-AAG-001 may encourage angiogenesis and inflammation, which would help endometriosis develop (Li et al., 2022d). Nonalcoholic fatty liver disease (NAFLD) is defined as an abnormal accumulation of fat in the liver that occurs in addition to alcohol and other known liver damage causes. NAFLD has a worldwide incidence of 25% (Younossi et al., 2016). The prevalence of NAFLD has steadily increased over the past few decades to become the most prevalent chronic liver disease (Abdelmalek, 2021). According to certain research, impaired autophagy has been linked to the beginning of NAFLD (Niture et al., 2021). The expression of tRF-3001b, which targets and suppresses the expression of the autophagy-related gene Prkaa1, was upregulated in the liver tissue of NAFLD mice. However, silencing tRF-3001b greatly improves the pathophysiology of the disease and decreases triglyceride and cholesterol levels (Zhu et al., 2020).

### 5.3 Treatment of disease

tRF-19-W4PU732S, produced from mature tRNA-Ser-AGA, targets the ribosome protein RPL27A in the breast cancer cell line MCF-7 cells to enhance cell proliferation, migration, invasion, and decrease apoptosis (Luo et al., 2023). In contrast, the reverse outcome is observed in MDA-MB-231 cells when tRF-19-W4PU732S is suppressed (Zhang et al., 2022). This discovery offers a fresh idea for breast cancer-targeted treatment. In recent years, the study of tsRNAs-related pharmacological treatment has progressively gained popularity. In ovarian cancer A2780 cells, tRF-T11 produced from Chinese yew displays anticancer efficacy similar to taxol but at a dosage that is 16 times lower. It targets the 3′UTRof TRPA1 mRNA via binding to the AGO2 protein (Cao et al., 2022). A tRNA-derived fragment from the 3′-end of tRNAGln (UUG), also known as HC83, is present in ginseng extract and has the ability to construct a 22-mer double-strand RNA. The production of VEGFA is subsequently upregulated as a result of this double-strand RNA’s direct downregulation of a lncRNA known as the myocardial infarction-associated transcript (MIAT). By targeting the lncRNA MIAT/VEGFA signaling pathway, HC83 can maintain the integrity of myocardial cytoskeleton and mitochondrial function and significantly improve myocardial function. The inhibitory effect on myocardial ischemia/reperfusion (MI/R) injury is 500 times stronger than metoprolol (Hu et al., 2022). Although TCM has been utilized preliminarily to treat ischemic heart disease, it is yet unknown how RNA research has been conducted in this sector. This finding will open up new possibilities for using RNA to cure diseases. The above studies reveal novel roles of plant-derived tsRNAs in disease therapy, which offers new hope for the creation of novel RNA medicines from nature.

### 5.4 Biomarker

tsRNA is more stable than other small RNAs because of its abundant methylation, terminal modifications and short length (Wang et al., 2020), which makes it difficult to be degraded by RNase. tsRNA is conserved between species (Chen et al., 2019; Yang et al., 2022b) and widely exists in human body fluids, such as blood, semen and leucorrhea (Nätt et al., 2019; Wu et al., 2021; Li et al., 2022d), so it can be applied as a novel non-invasive diagnostic and prognostic biomarker.

Endometriosis (EM) is a common sex hormone-dependent disorder, which is defined by the development of endometrial tissue outside the uterine cavity. Common clinical symptoms include dysmenorrhea, infertility, and chronic pelvic discomfort, affecting nearly 10% of women of childbearing age worldwide (Kvaskoff et al., 2021). At present, the diagnosis of EM is confirmed by pathological biopsy after surgical removal under laparoscopy. It is not only invasive but also may miss early disease lesions (Taylor et al., 2021). The serum marker CA125, often used in clinical practice to aid in the diagnosis of EM, has the drawbacks of low sensitivity and specificity. Li et al. sequenced exosomes from ectopic endometrial tissues and normal endometrial tissues and subsequently verified their expression by quantitative real-time PCR (qRT-PCR), and found that the content of tRF-Leu-AAG-001 in vaginal secretions of endometriosis patients was significantly higher than that of normal controls. They then used the receiver operating characteristic (ROC) to this data and discovered that the cutoff value is 0.3513, with an area under the curve (AUC) of 0.808 (Li et al., 2022d). This suggests that tRF-Leu-AAG-001 can better distinguish patients with EM from normal control groups. tRF-Leu-AAG-001 can therefore be employed as a potential non-invasive diagnostic biomarker to aid in the precise identification of EM. Gao et al. used the GEO database and TCGA database to screen out 11 differentially expressed tsRNAs between NSCLC patients and normal healthy people to establish a random forest prediction model. This model’s sensitivity, specificity and accuracy are all above 80%, which can be utilized to distinguish cancer patients from normal healthy people, and also has good diagnostic value (Gao et al., 2022).

The prognosis of the disease can also be predicted using tsRNA-based biomarkersts. According to Kaplan-Meier survival analysis, patients with colorectal cancer (CRC) who have higher levels of 5′-tiRNA-ProTGG have poorer disease-free survival (DFS) and overall survival (OS). Multivariate cox regression analysis highlighted that a high concentration of 5′-tiRNA-ProTGG is a poor prognostic factor for predicting short-term recurrence in CRC patients. It is independent of established prognostic factors in CRC (Tsiakanikas et al., 2022) (Table 2).

**TABLE 2:** Roles and applications of tsRNAs in diseases.

Aspect	Classification	Disease	Example of tsRNA(s)	Ref. Number
Occurrence of disease	Antitumor	Breast cancer	tRF3E	Falconi et al. (2019)
	Promote cancer	Breast cancer	ts-112	Farina et al. (2020)
		Gastric cancer	tRF-3019a	Zhang et al. (2020)
			tRF-3017A	Tong et al. (2020)
Progression of disease	Proliferation and apoptosis	Radiation-induced lung injury	tRF-Gly-GCC	Deng et al. (2022)
	Migration and invasion	Hepatocellular carcinoma	Gly-tRF	Zhou et al. (2021)
	Inflammation and angiogenesis	Endometriosis	tRF-Leu-AAG-001	Li et al. (2022d)
	Autophagy	Nonalcoholic fatty liver disease	tRF-3001b	Zhu et al. (2020)
Treatment of disease	Targeted therapy	Breast cancer	tRF-19-W4PU732S	Zhang et al. (2022)
	Plant-based drug therapy	Ovarian cancer	tRF-T11	Cao et al. (2022)
		Ischemic heart disease	HC83	Hu et al. (2022)
Biomarker	Diagnostic biomarker	Colorectal cancer	5′-tRF-GlyGCC	Wu et al. (2021)
		Endometriosis	tRF-Leu-AAG-001	Li et al. (2022d)
	Prognostic biomarker	Colorectal cancer	5′-tiRNA-ProTGG	Tsiakanikas et al. (2022)

## 6 Summary and outlook

Numerous advances have been made in understanding how tsRNA regulates the onset and progression of different diseases. Like microRNAs, tsRNAs can combine with AGO protein to form AGO/RISC complex and then target downstream genes through base pairing, leading to biological effects, such as gene silencing, regulation of ribosome processing and protein production. However, tsRNA is not limited to this mode of action. tsRNAs can also directly bind to proteins and participate in the regulation of gene transcription, protein modification, gene expression, protein stabilization and signaling pathways. Therefore, tsRNA has its own unique mode of action and is not a type of microRNA. By influencing the expression of tumor suppressor genes, tsRNA plays a role in the development of cancers. They can affect the progression of diseases not only by regulating cell proliferation or apoptosis, migration and invasion but also by affecting the formation of inflammatory cytokines, angiogenesis and autophagy. With the rise of precision medicine in follow-up, the clinical potential of tsRNA has also attracted extensive attention. Due to its involvement in the development of diseases through influencing different signaling pathways, tsRNA is expected to be a target for the treatment of diseases. Investigating whether tsRNA is expressed explicitly in tissue samples and selectively repressing tsRNA expression may be a promising strategy for disease therapy. With the development of plant-derived tsRNAs for disease treatment, plant extracts are expected to be used as drug therapy. Furthermore, tsRNA is more stable than other small RNAs due to its various modifications, so it can be utilized as a biomarker to predict the occurrence and prognosis of diseases. Applying biomarkers based on tsRNA will have broad application prospects in disease diagnosis. It may become a specific biomarker for molecular diagnosis of tumors and provide a non-invasive, safe, and reliable treatment method for clinical diagnosis and early screening of diseases. It also helps predict prognosis, monitor progression, and improve patient risk stratification. In the future, tsRNA may become a new specific biomarker of various diseases and be used as a marker for disease-targeted therapy and efficacy monitoring to prevent and treat diseases more comprehensively. Given the crucial potential application value of tsRNA, a systematic study of the mechanism of tsRNA in various conditions can help to form individualized medical treatment programs and improve the clinical outcomes of patients.

Although tsRNA is a research hotspot, it has not been fully explored. tsRNAs that have not been encoded in the tsRNA database may also have important biological functions, which need to be found by further research. The existing tsRNA database is very few, which causes difficulties in the research and analysis of tsRNAs. In many cases, it is necessary to use a microRNA database to study tsRNAs. In addition, the structure of tsRNAs and microRNAs are not the same. tsRNA is not a single-stranded RNA but contains part or complete stem-loop spatial structure. Due to technical limitations, the existing experimental means cannot synthesize the spatial structure of tsRNAs *in vitro*, which makes it difficult to further explore the function of tsRNAs. Although exogenous tsRNA without stem-loop spatial structure can show functional differences in cells, whether it has the same function as human tsRNA *in vivo* remains to be further explored. Finally, the use of tsRNA as a biomarker and pharmacological target should be the main emphasis of future research. The creation of particular medications that target tsRNA for various disorders has to be sped up by researchers. It is possible to obtain therapeutic tsRNA extracts from Chinese herbal remedies. Different tsRNA joints or tsRNA coupled with the clinical index of diagnosis and prognosis of artificial intelligence model should be energetically explored in recent years with the emergence of non-coding RNA biomarkers. I hope this review will provide help for the integration of tsRNAs into the clinic.

## Figures and Tables

**Table:** 

**tRNA**	transfer RNA
**tsRNA**	tRNA-derived small RNA
**tRFs**	tRNA-derived fragments
**AGO**	Argonaute
**UTR**	untranslated region
**TCM**	traditional Chinese medicine
**ANG**	Angiotensin
**tiRNAs**	tRNA-derived stress-induced RNAs
**RISC**	RNA-induced silencing complex
**CDS**	coding region
**OA**	osteoarthritis
**THBS1**	thrombospondin-1
**PBS**	primer binding site
**RNAi**	RNA interference
**ERV**	endogenous retroviruses
**NSCLC**	non-small cell lung cancer
**mESCs**	mouse embryonic stem cells
**PDAC**	pancreatic ductal adenocarcinoma
**hnRNP L**	heterogeneous nuclear ribonucleoprotein L
**LIF**	leukemia inhibitory factor
**IL-6**	interleukin-6
**IRES**	internal ribosome entry site
**RA**	retinoic acid
**PTC**	Papillary thyroid carcinoma
**GC**	gastric cancer
**PKM2**	pyruvate kinase M2
**NCL**	nucleolin
**RILI**	radiation-induced lung injury
**ROS**	reactive oxygen species
**EMT**	epithelial-mesenchymal transition
**NAFLD**	nonalcoholic fatty liver disease
**MIAT**	myocardial infarction-associated transcript
**MI/R**	myocardial ischemia/reperfusion
**EM**	endometriosis
**qRT-PCR**	quantitative real-time PCR
**ROC**	receiver operating characteristic
**AUC**	area under the curve
**CRC**	colorectal cancer
**DFS**	disease-free survival
**OS**	overall survival
